# Unmet need for contraception and its associated factors among adolescent and young women in Guinea: A multilevel analysis of the 2018 Demographic and Health Surveys

**DOI:** 10.3389/fgwh.2022.932997

**Published:** 2022-11-17

**Authors:** Sidikiba Sidibé, Fassou Mathias Grovogui, Karifa Kourouma, Delphin Kolié, Bienvenu Salim Camara, Alexandre Delamou, Seni Kouanda

**Affiliations:** ^1^ Institut Africain de Santé Publique (IASP/USTA), Saint Thomas D’Aquin University, Ouagadougou, Burkina Faso; ^2^ African Centre of Excellence in the Prevention and Control of Communicable Diseases (CEA-PCMT), Faculty of Sciences and Health Techniques, Gamal Abdel Nasser University, Conakry, Guinea; ^3^ Centre National Training and Research Centre in Rural Health of Maferinyah, Forécariah, Guinea

**Keywords:** unmet need, multilevel analysis, adolescents and young women, demographic health survey, Guinea, contraception

## Abstract

Despite the recent repositioning efforts to increase the use of modern contraceptives, the prevalence of unmet need for contraception remains high among adolescent and young women in Guinea. This study analyzed the individual and contextual factors associated with the unmet need for contraception among adolescent and young women in 2018 in Guinea. We conducted a secondary analysis of the 2018 Demographic and Health Survey data. Multilevel mixed-effects logistic regression models were used to assess the association between individual and contextual characteristics and unmet need for contraception among adolescents and young women. Adjusted odds ratios (AORs) with their 95% confidence intervals (CIs) were calculated, with statistical significance set at *p* < 0.05. The prevalence of total unmet need for contraception was 22.6% (95% CI, 18.1–27.8). Being an adolescent aged 15–19 years (AOR = 1.44; 95% CI, 1.01–2.05), unmarried (AOR = 5.19; 95% CI, 3.51–7.67), having one or two children (AOR = 3.04; 95% CI, 2.18–4.25), or more than two children (AOR = 4.79; 95% CI, 3.00–7.62) were individual factors associated with the unmet need for contraception. As for community factors, only living in Labé (AOR = 2.54; 95% CI, 1.24–5.18) or Mamou (AOR = 1.73; 95% CI, 1.21–2.48) was significantly associated with the unmet need for contraception. In conclusion, both individual and community characteristics were significantly associated with the unmet need for contraception. This highlights the need to focus and strengthen communication and counseling strategies targeting adolescents and young women and aiming to increase the uptake of family planning in Guinea.

## Introduction

Worldwide, 270 million women of reproductive age (15–49 years) had an unmet need for contraception in 2019; this unmet need is projected to increase by 10% by 2030 (1–3). Unmet need for contraception is defined as the proportion of fecund and sexually active women who do not want to become pregnant but are not using any form of contraception (4, 5). Moreover, 24% of women of reproductive age in low-income countries who wanted to avoid pregnancy could not use a modern method of contraception, with the majority of them living in sub-Saharan Africa (SSA) (6, 7). The main consequences of unintended early pregnancy among adolescents and young women include dropping out from school (8, 9), prolonged labor, preterm birth, stillbirths, neonatal deaths, and maternal and perinatal mortality (10). Though the use of modern methods of contraception can reduce maternal and child fatality, the majority of women of reproductive age in SSA are still at high risk of unsafe abortions and their related consequences (7, 11, 12). Furthermore, adolescent girls and young women (aged 15–19 years) represent one of the most burned groups by unmet need for contraception and unintended pregnancies in countries in SSA (6). In fact, more than one in five adolescent and young women has an unmet need for contraception in SSA (13, 14).

This high unmet need for contraception and its related consequences have been reported to be associated with several factors such as individual, sociocultural-, knowledge- (about contraceptive methods), and healthcare service-related factors (15–18). However, most of these studies mainly focused in general on women of reproductive age who were married, highlighting the need of evidence of the unmet need of contraception among adolescents and young women.

In Guinea, the prevalence of contraception among sexually active adolescent girls and young women (aged 15–24 years) using any contraceptive remains low along with a high fertility rate among adolescents (132 per 1,000 adolescents) and young people (205 per 1,000 women) (19). Despite the recent repositioning efforts to increase modern contraceptive prevalence in the country (20), more than 22% of women aged 15–49 years had an unmet need for contraception in Guinea (19). In fact, specific challenges exist in meeting the contraceptive needs of female adolescents and young women. These challenges include, among others, the non-adaptability of sexual and reproductive health services for adolescents, particularly those of family planning (FP), as well as not prioritizing the concerns of adolescent and young women with regard to contraception in available policy (21). This highlights the knowledge gaps of individual and community factors associated with the unmet need for contraception among adolescent and young women in Guinea. Therefore, the aim of the present study was to analyze the individual and community factors associated with the unmet need for contraception among adolescents and young women in Guinea in 2018. This information could be used as evidence for guiding policy and interventions on sexual and reproductive health targeting adolescents and young women.

## Materials and methods

### Data and population

Data were extracted from the 2018 Guinean Demographic and Health Surveys (DHS 2018). The DHS are nationally representative demographic and household surveys that collect data on a wide range of reproductive, maternal, and child health topics, such as fertility, health-seeking behaviors, and FP methods. A two-stage stratified cluster design was applied in survey sampling based on a list of enumeration areas (EAs) of the 2018 General Population Census of the Republic of Guinea. This study sample included sexually active adolescent and young women aged 15–24 years at the time of the survey. The survey covered the populations living in the strata of Guinea's eight administrative regions (Conakry, Boke, Faranah, Kankan, Kindia, Labe, Mamou, and N’Zérékoré) in Guinea. Women with missing data on the outcome variable were excluded from the analyses.

### Definition of variables

#### Outcome variable

The outcome coded in binary (1 = Yes for unmet need and 0 = No for not unmet need) was generated from a constructed the DHS dataset. Unmet need for contraception was defined as women who do not want to become pregnant but are not using any contraception among all sexually active adolescents and young women (4, 22). Those women include married and/or sexually active unmarried considered fecund but neither pregnant nor in postpartum amenorrhea, and willing to delay their next birth by at least 2 years or limit their pregnancies without using a modern method of FP. This definition includes those currently pregnant or in postpartum amenorrhea but whose current pregnancy or last birth was not desired (4, 22).

#### Independent variables

Individual- and community-level variables were considered as determinants of the unmet need for contraception for this analysis based on a literature review and in line with our multilevel analytic approach.

Individual-level variables included socio-demographic characteristics (age, level of education, marital status, quintile wealth number of living children, knowledge of modern contraceptive, and exposure to FP messages). For the need of our analyses, the participants' age was recoded into two age groups (15–19 years and 20–24 years). The women's level of education was also recoded into no formal education, primary, secondary, and higher level of education. The participants' marital status was defined as currently in union (married or living in the union) and not in union (single, divorced or widowed, or separated). In the past 6 months, exposure to family planning messages was defined as recalling a family planning message heard or seen from the media, including radio, television, or newspapers.

The women's economic status was measured through the household wealth index as defined in the DHS dataset. The DHS wealth index was calculated using economic status indicators that included the main assets available at the household level at the time of the survey. Wealth index quintiles were defined for each household (poorest, poor, middle, richer, and richest).

Community-level variables included residence (rural and urban), administrative region (Conakry, Kindia, Boké, Labé, Mamou, Faranah, Kankan, N'Zérékoré), religion (Muslim and Christian/Other), and ethnicity (Soussou, Peulh, Malinké, and Others).

### Statistical analysis

The data were processed and analyzed using Stata version 16.1. Two levels of statistical analysis applied to the data. At the first level, we described individual and community characteristics and then obtained absolute numbers and weighted percentages of explanatory variables and the prevalence of unmet need for contraception among adolescent and young women. The purpose of weighting the sample data was to improve its representativeness regarding the study population’s size, distribution, and characteristics. We then described the prevalence of unmet need among adolescent and young women.

The second level of analysis consisted of using multilevel modeling to assess adolescents and young women’s individual and community characteristics associated with the unmet need for contraception. Before constructing our models, we first evaluated the collinearity between the independent variables. The purpose was to determine the suitability of the variables selected for inclusion in the analyses. As a rule of thumb in regression analyses, a mean Variance Inflation Factor (VIF) score <5 is tolerated. There was no multicollinearity (all VIF < 5). In contrast, a mean VIF score of 5–10 suggests that the regression coefficients could be mis-estimated (23). Mixed-effects logistic regression (fixed and random) was used for the multivariate analyses to explore the explanatory variables. Fixed effects were estimated using the logistic regression's adjusted odds ratio (AOR), and random effects were estimated using the intra-cluster correlation coefficient (ICC).

Four models were constructed. Model 0 included no independent variables. Model 1 had only individual-level characteristics, while Model 2 was based solely on community-level characteristics (cluster). Finally, a multilevel model (Model 3) was constructed by including the individual- and community-level characteristics. All analyses incorporated design adjustment using sampling weights, clustering, and stratification. Adjusted odds ratios (AORs) measuring the associations between the unmet need for contraception and various covariates were then calculated. A threshold of 5% was considered for all statistical analyses along with a 95% confidence interval (CI).

## Results

### Individual and community characteristics

**Figure 1:**
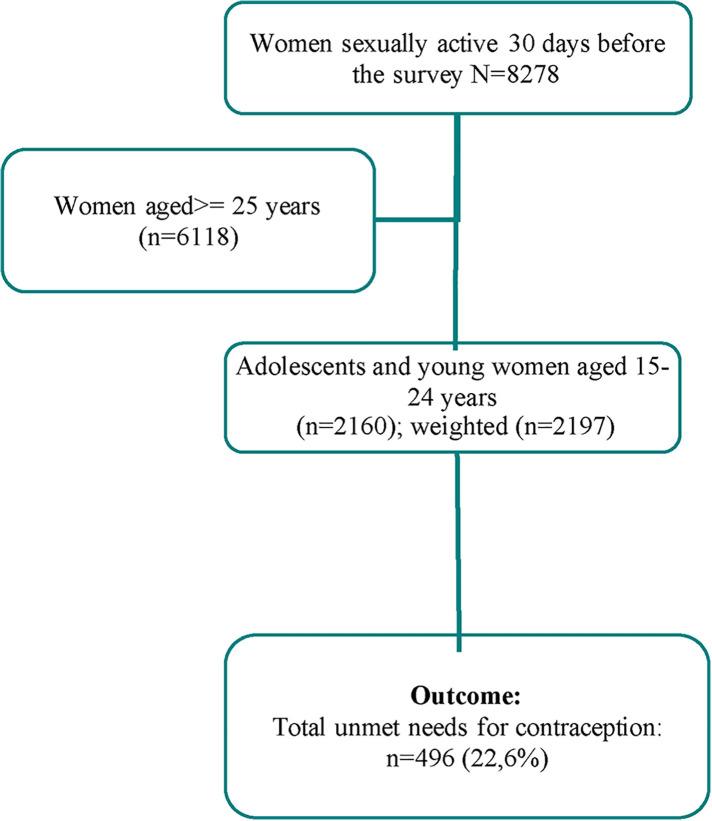
Flow chart of total number of adolescents and young women (aged 15–24), who were sexually active included in the analysis, 2018 Guinea.

Table 1 presents the individual and community characteristics of the study participants. A total of 2,197 sexually active adolescents and young women were included in the analyses (Figure 1). Of these, 1,310 (59.6%) were aged 20–24 years with the majority not currently married (86.3%) and with no formal education (63.4%). Approximately 7 out of 10 (67.0%) participants reported not being exposed to FP messages in the media. However, more than four-fifths (86.9%) reported being familiar with at least one modern method of contraception.

**Table 1:** Socio-demographic characteristics Adolescent and Young women (aged 15–24), 2018, Guinea.

	*n* = 2197	%	%95 CI	
			Lower	Upper
Individuals				
Age group at the time of the survey				
15–19	887	40.4	38.0	42.8
20–24	1310	59.6	57.2	62.0
Education level				
No formal education	1395	63.4	60.1	66.7
Primary	347	15.8	13.9	17.9
Secondary/higher	445	20.8	18.3	23.4
Marital status				
Not married	301	13.7	11.8	15.9
Married	1896	86.3	84.1	88.2
Number of living children that the women have				
0	739	33.6	33.0	36.3
1_2	1246	56.7	54.2	59.2
3+	212	9.7	8.4	11.1
Wealth index group				
Poorest	436	19.9	17.0	23.0
Poorer	445	20.3	17.8	23.1
Middle	442	20.1	17.9	22.7
Richer	480	21.9	18.8	25.3
Richest	394	17.9	15.3	20.9
Exposed to FP messages through media over the last few months				
Not exposed	1473	67.0	63.5	70.4
Exposed	724	33.0	29.6	36.5
FP knowledge				
No	287	13.1	11.1	15.4
Yes	1910	86.9	84.6	88.9
Contextual variables				
Residence area				
Urban	757	34.4	29.6	39.7
Rural	1441	65.6	60.3	70.4
Administrative region				
Boké	190	8.6	7.3	10.2
Conakry	319	14.5	12.2	17.2
Faranah	237	10.8	8.8	13.2
Kankan	409	18.7	15.3	22.5
Kindia	309	14.1	12.1	16.3
Labé	206	9.4	7.8	11.3
Mamou	167	7.6	6.0	9.7
Nzérékoré	360	16.4	14.3	18.8
Ethnic group				
Soussou	429	19.5	16.5	22.9
Peulh	733	33.4	29.6	37.3
Malinke	699	31.8	27.8	36.2
Forestier/other	336	15.3	12.5	18.6
Religion				
Muslim	1891	86.0	82.8	88.7
Christian and other	306	14.0	11.3	17.2

The participants' community-level characteristics are also presented in Table 1. Overall, 65.6% of the adolescent and young women lived in rural areas, and 86.0% were Muslim.

### Prevalence of total unmet need

The overall prevalence of unmet need for contraception among adolescents and young women aged 15–24 years was 22.6% (95% CI, 18.1–27.8). This prevalence was statistically significantly higher among adolescents and unmarried young women (38.5% vs 20.1%; *p* < 0.001). Other individual and community characteristics that appeared to have a higher prevalence of unmet need for contraception included the number of living children (*p* = 0.021) and administrative region (*p* < 0.001) (Table 2).

**Table 2:** Prevalence of total unmet need among sexually active old adolescent and young women aged 15–24 (*n* = 2197).

Characteristics	*N*	%	95% CI		*p*-Value
			Lower	Upper	
Individual factors					
Age group at the time of the survey	** **				0.479
15–19	887	23.5	18.8	28.9	
20–24	1310	21.9	16,8	28.1	
Education level	** **				0.099
No formal education	1395	21.0	15.7	27.6	
Primary	347	28.7	22.4	35.9	
Secondary/higher	455	22.7	18.0	28.0	
Marital status	** **				0.001
Not married	301	38.5	32.0	45.2	
Married	1896	20.1	15.5	25.5	
Number of living children that the women have					
0	739	17.8	13.1	23.8	0.0208
1_2	1246	24.5	18.9	31.0	
3+	212	27.9	21.0	35.9	
Wealth index group	** **				0.569
Poorest	436	22.3	13.6	34.3	
Poorer	446	19.5	14.5	25.5	
Middle	441	23.0	14.9	33.8	
Richer	480	25.1	20.	30.9	
Richest	394	22.8	19.9	26.1	
Exposed to FP messages through media over the last few months	** **				0.118
Not exposed	1473	23.7	18.2	30.2	
Exposed	724	20.3	16.8	24.3	
FP knowledge	** **				0.514
No	287	24.7	16.3	35.6	
Yes	1910	22.3	17.9	27.4	
Contextual variables					
Residence area					
Urban	757	23.5	20.3	27.1	0.729
Rural	1440	22.1	14.4	30.6	
Administrative region					0.018
Boké	189	20.8	7.8	44.9	
Conakry	319	24.5	24.5	24.6	
Faranah	237	14.9	12.0	18.2	
Kankan	410	14.0	10.8	18.0	
Kindia	309	23.3	20.1	26.8	
Labé	206	35.7	28.2	43.8	
Mamou	167	28.1	26.3	27.0	
Nzérékoré	360	26.0	22.3	30.1	
Ethnic group					0.205
Soussou	429	25.5	20.5	31.3	
Peulh	733	23.8	15.4	35.0	
Malinke	699	17.8	12.4	24.9	
Forestier/other	337	25.9	23.5	28.6	
Religion					0.219
Muslim	1890	22.0	16.9	28.0	
Christian and other	307	26.4	24.5	28.3	
Total unmet need	496	22.6	18.1	27.8	

### Multivariate analysis

Model 3 included both individual- and community-level characteristics (Tables 3, 4).

**Table 3:** Bivariate and multilevel logistic analysis of factors associated with total unmet need among sexually active old adolescent and young women aged 15–24—Guinea.

Characteristics	Bivariate analysis				Multilevel analysis (Model 3)			
	OR	95% CI		*p*-Value	AOR	95% CI		*p*-Value
		Lower	Upper			Lower	Upper	
**Age group at the time of the survey**								
15–19	1.09	0.85	1.39	0.491	1.44	1.01	2.05	**0**.**044**
20–24	Ref				Ref.			
Education level								
No formal education	0.91	0.67	1.24	0.548	1.10	0.73	1.65	0.599
Primary	1.37	0.95	1.99	0.092	1.36	0.98	1.90	0.068
Secondary/higher	Ref				Ref.			
**Marital status**								
Not married	2.49	1.79	3.46	<0.001	5.19	3.51	7.67	**<0**.**001**
Married	Ref							
**Number of living children**								
0	Ref				Ref.			
1_2	1.49	1.15	1.94	0.003	3.04	2.18	4.25	**<0**.**001**
3+	1.78	1.20	2.63	0.004	4.79	3.00	7.62	**<0**.**001**
Wealth index group								
Poorest	0.97	0.66	1.43	0.873	0.73	0.48	1..12	0.128
Poorer	0.82	0.55	1.21	0.312	0.58	0.32	1.06	0.069
Middle	1.01	0.70	1.46	0.956	0.74	0.46	1.22	0.198
Richer	1.13	0.77	1.65	0.529	1.09	0.72	1.67	0.637
Richest	Ref				Ref.			
Exposed to FP messages through media over the last few months								
Not exposed	1.22	0.95	1.58	0.125	1.23	0.99	1.53	0.062
Exposed	Ref				Ref			
FP knowledge								
No	1.14	0.82	1.59	0.423	1.28	0.89	1.84	0.150
Yes	Ref				Ref			
Contextual variables								
Residence area								
Urban	Ref				Ref.			
Rural	0.92	0.72	1.17	0.509	1.28	0.45	1.99	0.863
**Administrative region**								
Conakry	Ref				Ref			
Boké	0.81	0.51	1.27	0.353	0.78	0.23	2.64	0.643
Faranah	0.54	0.34	0.83	0.006	0.58	0.29	1.16	0.104
Kankan	0.49	0.30	0.81	0.005	0.44	0.28	0.67	**0**.**002**
Kindia	0.93	0.61	1.41	0.737	0.87	0.71	1.07	0.154
Labé	1.70	1.06	2.70	0.023	2.54	1.24	5.18	**0**.**018**
Mamou	1.20	0.81	1.77	0.357	1.73	1.21	2.48	**0**.**009**
Nzérékoré	1.08	0.74	1.58	0.698	0.96	0.68	1.36	0.788
Ethnic group								
Soussou	1.58	1.11	2.24	0.010	1.00	0.80	1.25	0.976
Peulh	1.44	1.06	1.96	0.020	0.70	0.46	1.07	0.087
Malinke	Ref	Ref			Ref			
Forestier/other	1.61	1.13	2.31	<0.001	1.05	0.47	2.35	0.881
Religion								
Muslim	Ref				Ref			
Christian/other	1.27	0.94	1.71	0.114	0.95	0.45	1.99	0.863

Bold values indicate evidence statistically significant.

**Table 4:** Multilevel logistic analysis of factors associated with total unmet need among sexually active old adolescent and young women aged 15–24—Guinea.

Characteristics	Null model				Model 1				Model 2				Model 3			
	AOR	95% CI		*p*-Value	AOR	95% CI		*p*-Value	AOR	95% CI		*p*-Value	AOR	95% CI		*p*-Value
		Lower	Upper			Lower	Upper			Lower	Upper			Lower	Upper	
Measure of variation																
Cluster variance		0.20			0.24				0.02				0.016			
Explained variance (PCV %)	Reference				22.5				88.4				91.5			
The intra-cluster correlation coefficient (ICC) in %	5.64				6.83				0.69				0.51			
**Age group at the time of the survey**																
15–19					1.38	0.98	1.95	0.062					1.44	1.01	2.05	0.044
20–24					Ref.								Ref.			
Education level																
No formal education					1.05	0.67	1.64	0.798					1.10	0.73	1.65	0.599
Primary					1.40	0.97	2.01	0.064					1.36	0.98	1.90	0.068
Secondary/higher					Ref.								Ref.			
**Marital status**																
Not married					5.38	3.54	8.14	<0.001					5.19	3.51	7.67	<0.001
Married																
**Number of living children**																
0					Ref.								Ref.			
1_2					3.12	2.19	4.44	<0.001					3.04	2.18	4.25	<0.001
3+					4.69	2.91	7.57	<0.001					4.79	3.00	7.62	<0.001
Wealth index group																
Poorest					0.92	0.50	1.70	0.762					0.73	0.48	1..12	0.128
Poorer					0.76	0.49	1.17	0.175					0.58	0.32	1.06	0.069
Middle					0.96	0.51	1.81	0.884					0.74	0.46	1.22	0.198
Richer					1.13	0.80	1.61	0.414					1.09	0.72	1.67	0.637
Richest					Ref.								Ref.			
Exposed to FP messages through media over the last few months																
Not exposed					1.23	0.94	1.63	0.110					1.23	0.99	1.53	0.062
Exposed					Ref								Ref			
FP knowledge																
No					1.27	0.84	1.93	0.213					1.28	0.89	1.84	0.150
Yes					Ref								Ref			
Contextual variables																
Residence area																
Urban									Ref.				Ref.			
Rural									0.93	0.73	1.20	0.570	1.28	0.45	1.99	0.863
**Administrative region**																
Conakry									Ref.				Ref			
Boké									0.89	0.27	2.94	0.280	0.78	0.23	2.64	0.643
Faranah									0.58	0.39	0.86	0.836	0.58	0.29	1.16	0.104
Kankan									0.48	0.33	0.70	0.014	0.44	0.28	0.67	0.002
Kindia									0.95	0.78	1.17	0.002	0.87	0.71	1.07	0.154
Labé									2.54	1.37	4.71	0.619	2.54	1.24	5.18	0.018
Mamou									1.74	1.24	2.45	0.009	1.73	1.21	2.48	0.009
Nzérékoré									0.92	0.56	1.50	0.784	0.96	0.68	1.36	0.788
Ethnic group																
Soussou									1.06	0.89	1.26	0.448	1.00	0.80	1.25	0.976
Peulh									0.65	0.44	0.94	0.029	0.70	0.46	1.07	0.087
Malinke									Ref				Ref			
Forestier/other									1.06	0.47	2.42	0.863	1.05	0.47	2.35	0.881
Religion																
Muslim									1.12	0.51	2.42	0.743				
Christian/other									Ref.				0.95	0.45	1.99	0.863

Bold values indicate evidence statistically significant.

After adjusting for individual- and community-level variables, the participants who were adolescents aged 15–19 years (AOR = 1.44; 95% CI, 1.01–2.05), not married (AOR = 5.19; 95% CI, 3.51–7.67), had one to two children (AOR = 3.04; 95% CI, 2.18–4.25) or had more than two children (AOR = 4.79; 95% CI, 3.00–7.62), from Labe (AOR = 2.54, 95% CI, 1.24–5.18) or from Mamou (AOR = 1.73, 95% CI, 1.21–2.48), compared to their counterparts, were more likely to have an unmet need for contraception (Table 3).

Introducing both individual- and community-level characteristics in the model reduced the ICC by 9.15% [(0.196931–0.0169404) * 100/0. 0.196931]. Therefore, the study population characteristics included in our final model explain around 92% of the individual variation in unmet need among adolescents and young women in Guinea (Table 4).

## Discussion

The study shows that more than one-fifth of adolescents or young women experienced unmet need for FP in Guinea in 2018. It also shows the higher prevalence among adolescents and unmarried young women, and the unmet need increased with the number of children had by women. The main factors associated with unmet need for contraception among the study population were age, marital status, number of children and administrative region.

The high prevalence (22.6%) of unmet need for contraception among our study participants is one of the factors hampering the achievement of the Sustainable Development Goals, i.e., reducing maternal and neonatal mortality and morbidity. Our results are consistent with those of Malawi (22.0%) (13), Mali (21.4%), and Burkina Faso (23.9%) (14). The observed results can be explained in part by a disparity in access to modern methods of contraception. Access to maternal, reproductive, and child health services was negatively affected by the Ebola epidemic in West Africa between 2014 and 2016 (24–25). Despite this, the country has developed a national plan to reposition FP (2014–2018) to improve access to and use of modern methods of FP, particularly among adolescents and young women (26). A budgeted national FP plan for Guinea 2019–2023 was also developed in 2018 to support these efforts. The plan focuses on FP awareness campaigns in the media and schools, integration of FP services into maternal health services (postabortion care and immunization services), and training of maternal health providers (21). Beyond such efforts, this study reveals the importance of supporting intervention plans adapted to each region and district's local context, emphasizing community engagement and participation, including men as partners.

In our study, adolescents aged 15–19 years were more likely to have an unmet need for contraception than young women aged 20–24 years. Our results confirm the findings reported in other studies conducted in Ghana, Ethiopia, and Zambia (27–30). These results could be explained by the fact that young women generally live in union with the desire to have children. In contrast, adolescent girls typically face stigma in communities about their sexual behavior when using health services outside of marriage. The strategy of improving community awareness and facilitating access to methods of contraception by considering women's real needs is essential to increasing the use of contraception among women in general. However, it is necessary to promote a dialogue between parents and their children on sexuality and the benefits of methods of contraception for birth control.

Marriage and the number of children are two critical aspects of community life in Africa. In our study, these two factors were statistically significantly associated with the unmet need for contraception. Adolescents and unmarried young women were more likely to have an unmet need for contraception. Even more, adolescents and young women who had one or two children and those who had three or more children were three and four times, respectively, more likely to have an unmet need for contraception. These women certainly do not need to limit their number of children. While Guinean women desire, on average, four children, the DHS 2018 (19) showed that the average number of children born alive among adolescents and young women is approximately one and two, respectively. Evidence from Ethiopia shows that the unmet need for contraception among these women are more for birth spacing (31). In the context where the newly married woman must demonstrate her fertility (32), adolescents and young women ever married would like to space out their last birth or current or next pregnancy by a few months or years. This explains the increase in unmet need for contraception with the number of children among the study population. Spacing births is a way for women to organize their socioeconomic life and their motherhood. Some studies report that nearly half of young married women in sub-Saharan Africa have an unmet need for contraception (31, 33–35). Reducing the unmet need for modern FP methods among adolescents and young women requires, therefore, local approaches that address married women’s access to methods of contraception that meet their birth spacing needs. In addition, the involvement of partners as key actors in the strategy will ensure durability.

Our results do not confirm that exposure to FP messages through the media significantly influences the unmet need for contraception among adolescents and young women. Indeed, participants not exposed to FP messages through the media were not significantly associated with the unmet need for contraception. Contrary results have been reported by other studies that found that women not exposed to media or counseling were more likely to have an unmet need for contraception than other women (12, 36–39).

We also found no statistical relationships between adolescents' level of education and young women's unmet need for contraception as expected. In fact, many studies in Africa show that women's use of contraception increases with her level of education (28, 30, 40, 41), particularly among adolescent girls (42–44). The lack of a statistical relationship observed in our study could be explained by more important factors such as the supply of methods of contraception, the costs associated with adopting a method of contraception, or the weight of community values that most influence access to and use of FP methods. In addition, our results show that more than half of the adolescents and young women had no formal education. The relatively low proportion of formally educated adolescents and young women has implications for their access to and use of health information. Therefore, efforts for reducing the unmet need for contraception among adolescents and young women should include improving women's access to education in general, resulting in their financial empowerment and strengthening their decision-making capacity regarding their fertility and better access to health information (31).

### Strengths

This study has some strengths that need to be highlighted. It used a nationally representative sample that accounts for complex sampling procedures to examine the individual and community factors associated with the unmet need for contraception among adolescents and young women. In addition, it used a multivariable model that considers differences across communities to adjust for potential confounding factors. These findings could help the Ministry of Health and its partners to support public health interventions to improve adolescents and young women's reproductive health in Guinea. They also point to challenges to address in using modern methods of contraception among this targeted group.

### Limitations

The present study has some limitations. First, the temporal relationship between the unmet need for FP among the study population with the covariate could not be established. Second, this study was solely based on variables related to women’s individual and community characteristics but did not include information related to the availability, accessibility, and quality of FP services for adolescents and young women. Moreover, the study did not analyze information related to the study population’s previous experiences of abortions, neonatal mortality, or adverse outcomes, for instance, which may highly influence their need for contraception.

## Conclusion

The overall prevalence of unmet need for contraception remains high among adolescents and young women in Guinea. This study revealed individual- and community-level factors associated with the unmet need for contraception among this sexually active population group. These findings suggest that communication strategies for adolescents and young women to reduce the unmet need for contraception should emphasize sexual education and awareness campaigns through the media and venues where young women gather. In addition, individual counseling in sexual and reproductive health services should be better directed toward adolescents and young married women already having children, considering their individual and contextual characteristics.

## Data Availability

Publicly available datasets were analyzed in this study. These data can be found here: https://dhsprogram.com/data/available-datasets.cfm.
